# Vitamin K deficiency-induced hemorrhagic shock after thoracentesis: a case report

**DOI:** 10.1186/s12876-019-0978-0

**Published:** 2019-04-18

**Authors:** Hideya Itagaki, Takuro Hagino

**Affiliations:** Honjoudaiichi Hospital, 110, Iwabuchishita, Yurihonnjou, Akita, 015-8567 Japan

**Keywords:** Vitamin K deficiency, Hemorrhagic shock, Cholecystitis, Intercostal artery, Thoracentesis

## Abstract

**Background:**

Vitamin K deficiency results in serious coagulation dysfunction, but hemorrhagic shock is rare. Herein, we describe a case of vitamin K deficiency and abnormality in the path of the intercostal artery, the combination of which led to hemorrhagic shock.

**Case presentation:**

An 83-year-old woman was hospitalized for suspected gallstones. She developed septic shock after 4 days of hospitalization. We considered cholecystitis or cholangitis and performed abdominal ultrasonography, which revealed gallbladder enlargement, biliary sludge, and hyperplasia of the bile duct wall. Antibiotic treatment with sulbactam/ampicillin (SBT/ABPC) was initiated on day four, and percutaneous transhepatic gallbladder drainage (PTGBD) was performed on day five. The treatment was successful, but the patient developed bilateral pleural effusion because of hypoalbuminemia. We performed drainage for bilateral pleural effusion on days 13 and 17. The patient developed hypotension on day 18; blood tests showed anemia and severe coagulation dysfunction but a normal platelet count. We suspected vitamin K deficiency-induced coagulation dysfunction because of previous antibiotic treatment and restricted diet, and it led to hemorrhagic shock. Massive right hemothorax was observed by computed tomography, and urgent interventional radiology was performed. We observed no injury to the intercostal artery truncus but confirmed an abnormality in the course of the intercostal artery; therefore, we inferred that the cause of hemothorax in this case was injury to a small vessel, not truncus because of the abnormality. Because of the likelihood of rebleeding, we performed coil embolization from the seventh to the ninth intercostal artery. Because we confirmed vitamin K deficiency-induced coagulation dysfunction, we referred to the concentration of protein induced by vitamin K absence/antagonist-II (PIVKA-II), and it was found to increase by 23,000.

**Conclusions:**

A combination of vitamin K deficiency and abnormality in the course of the intercostal artery led to hemorrhagic shock. When using certain antibiotics and restricting diet, it is important to measure coagulation function, even if the platelet count is normal. Further, when thoracentesis is performed, abnormalities in the course of the intercostal artery should be identified. Thoracentesis with ultrasound may prevent hemothorax.

## Background

Vitamin K is a fat-soluble vitamin. Fat malabsorption leads to secondary vitamin K deficiency. Causes of fat malabsorption include bile tract obstruction, intestinal mucosal dysfunction, and hepatic failure. Other causes include deficient fat intake and use of broad-spectrum antibiotics [1]. Vitamin K deficiency causes bleeding in various organs [2]. However, reports of vitamin K deficiency leading to hemorrhagic shock are rare. In the present case, when treating cholecystitis, we found that a combination of antibiotic treatment and restricted fat diet led to vitamin K deficiency and hemorrhagic shock.

## Case presentation

**Fig. 1:**
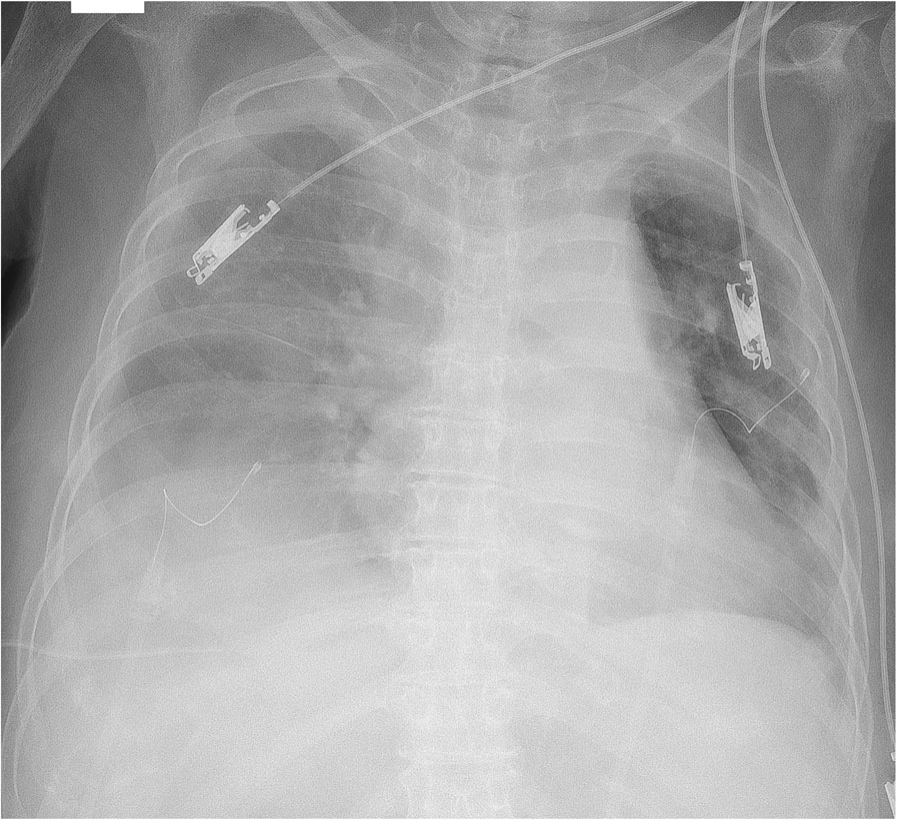
Draining of the left pleural cavities on day 17

**Fig. 2:**
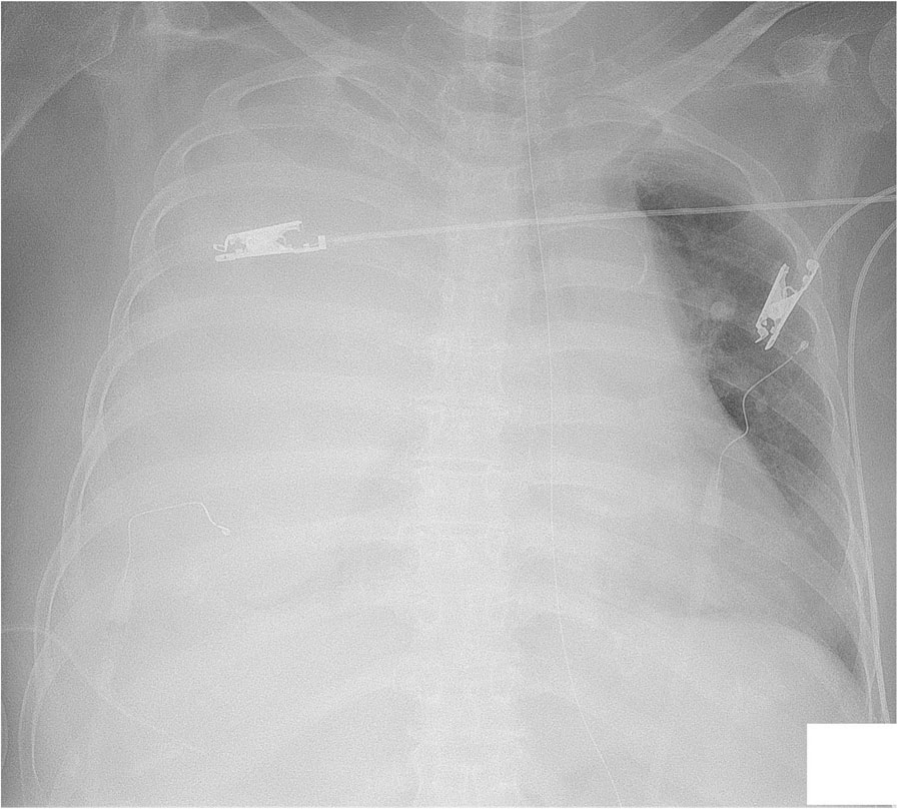
Massive hemothorax on day 18

**Fig. 3:**
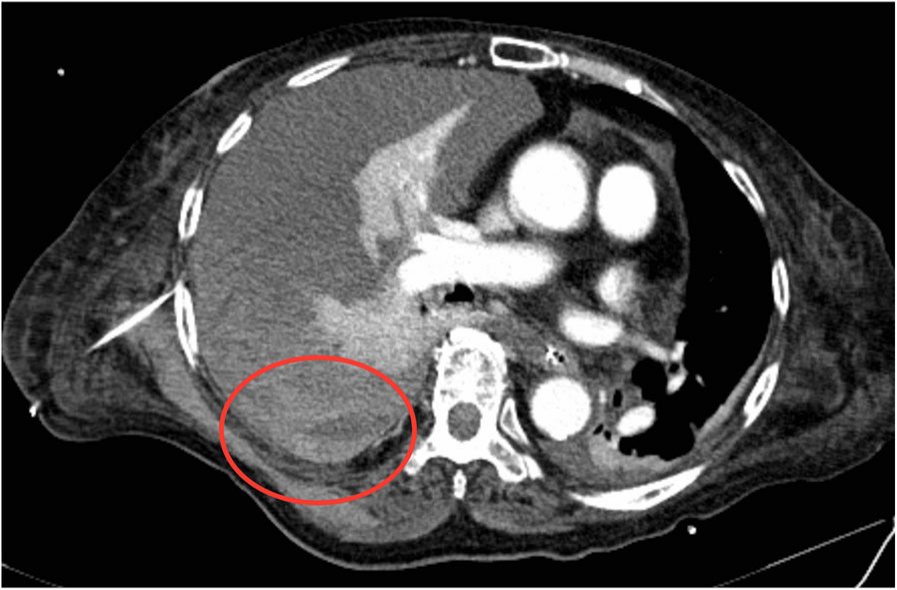
Enhancement rose from the 8th to the 11th intercostal(Red circle)

**Fig. 4:**
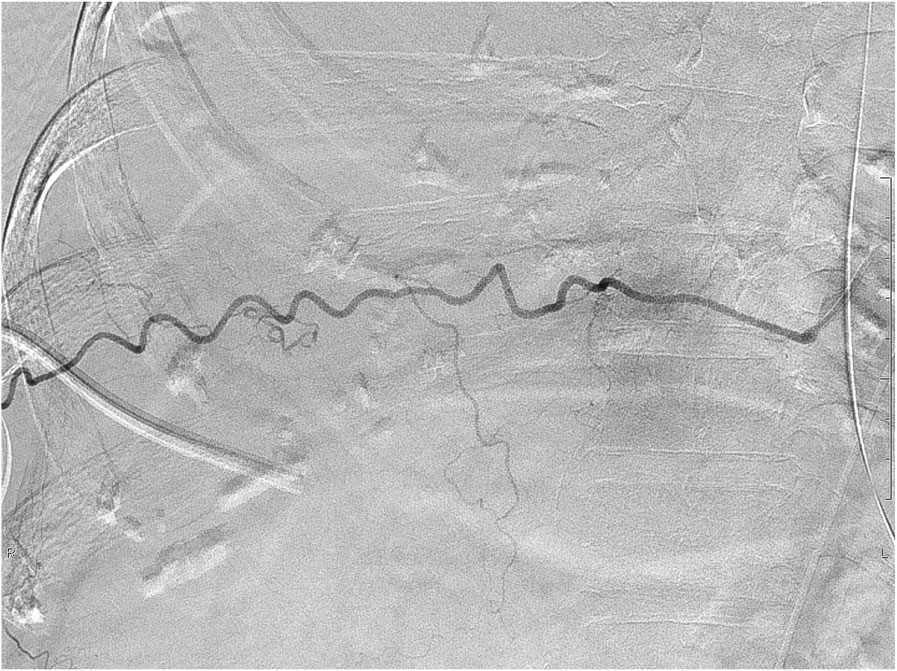
Abnormality in the path of the intercostal artery

An 83-year-old woman with epigastric pain was hospitalized for suspected gallstones. The patient’s medical history included diabetes, hypertension, hyperlipidemia, and dementia from stroke. The symptoms of epigastric pain disappeared after admission, but she developed a fever on day 2. Blood examination on day 4 revealed an inflammatory reaction (white blood cells, 12,200/μL; C-reactive protein, 27.39 mg/dL) and increased hepatobiliary enzymes (total bilirubin, 4.4 mg/dL; aspartate transaminase, 31 U/L; alanine transaminase, 54 U/L; lactate dehydrogenase, 217 U/L; alkaline phosphatase, 494 U/L; and gamma glutamyl transferase, 53 U/L). Urinalysis showed bilirubinuria. We considered cholecystitis or cholangitis and performed abdominal ultrasonography, which revealed gallbladder enlargement, biliary sludge, and hyperplasia of the bile duct wall. However, biliary expansion was not evident. Gallstone-related cholecystitis with bile duct inflammation was diagnosed. Antibiotic treatment with SBT/ABPC was initiated on day 4, and PTGBD was performed on day 5. The patient developed hypotension on day 6, and we therefore began noradrenaline administration. The disseminated intravascular coagulation did not merge (platelet count, 10.9 × 10^4^/μL; prothrombin time(PT), 11.4 s; activated partial thromboplastin time(APTT), 31.9 s). The treatment was successful, and the noradrenaline was discontinued on day 8. However, the patient developed bilateral pleural effusion because of hypoalbuminemia. We were unable to discontinue oxygenation. Therefore, we drained the right and left pleural cavities on days 13 and 17 (Fig. 1), respectively. The thoracentesis decided a puncture position in an echo, but we did not use the echo at the time of puncture. No signs of vascular injury that may have caused the hypotension were found. There was pleural effusion discharge of 300–400 ml from both drains, and the pleural effusion were transudative pleural effusion. On the morning of day 18, the patient had tachycardia (pulse rate, 130 beats/min) and hypotension (systolic blood pressure, 50 mmHg). We confirmed the presence of severe anemia using a blood test, and hemorrhagic shock was diagnosed. Because bloody fluid was exiting the drain in the right thoracic cavity at the time and we were unable to examine the right lung using chest X-ray, we suspected that the hemorrhagic shock was due to massive hemothorax (Fig. 2). A blood test showed extensive coagulation dysfunction (PT, 68.0; APTT, immeasurable), and we considered the presence of vitamin K deficiency because the platelet level was normal. We performed red blood cell and fresh frozen plasma transfusions, and administered vitamin K to improve the anemia and coagulation disorder as quickly as possible (PT, 11.5; APTT, 32.0). On day 19, we performed cystography–computed tomography to identify the bleeding source. Enhancement rose from the 8th to 11th intercostal space during the arterial phase, and we considered this to be the bleeding site (Fig. 3). We performed contrast-enhanced examination of the intercostal arteries during urgent interventional radiology. We were unable to clearly identify the bleeding source, but confirmed an abnormality in the path of the intercostal artery (Fig. 4). We inferred that the cause of hemothorax in this case was injury to a small vessel, and not truncus due of the abnormality. Because of the likelihood of rebleeding, we performed coil embolization from the seventh to the ninth intercostal artery. Subsequently, because we confirmed vitamin K deficiency-induced coagulation dysfunction, we measured the concentration of PIVKA-II, and it was found to increase with 23,000**.** The patient’s condition stabilized, and she was awaiting hospital transfer at the time of this writing.

## Discussion

Vitamin K deficiency causes bleeding in various organs, mucosal bleeding, bloody bowel discharge, and hematuria [2]. The absence of vitamin K affects the composition of coagulation factors II, VII, IX, and X and prolongs PT and APTT. However, other causes of PT, and APTT prolongation must be considered, such as hepatic failure and disseminated intravascular coagulation. Our patient was not believed to have hepatic failure because hepatic failure is associated with increase in liver enzymes and symptoms of hepatic failure. Because her platelet level was normal and her coagulation dysfunction normalized immediately upon transfusion, she was also not thought to have disseminated intravascular coagulation. Because high levels of PIVKA-II, an abnormal protein, were found and vitamin K deficiency was high, vitamin K deficiency was diagnosed.

Fat malabsorption, deficient fat intake, and use of broad-spectrum antibiotic treatment can lead to vitamin K deficiency. Particularly, use of antibiotics with an N-methyl-tetrazole-thiol group including sulbactam/cefoperazone, cefamandole, cefmetazole, and cefotetan leads to vitamin K deficiency [1]. In the present case, the patient underwent long-term fasting because of cholecystitis; antibiotic treatment was performed with SBT/ABPC, which led to vitamin K deficiency. Coagulation disorder due to cholecystitis is rare, but one such case report has been published [3]. Because we discontinued the patient’s diet, the patient was not supplemented with vitamin K. SBT/ABPC does not have an N-methyl-tetrazole-thiol group and therefore is not expected to cause vitamin K deficiency, but one case report has described vitamin K deficiency in association with SBT/ABPC [4]. Although this is considered a rare cause of vitamin K deficiency, one study showed that patients entering the intensive care unit present with vitamin K deficiency [5].

Pneumothorax, hemothorax, and hypotension are the main complications associated with thoracentesis. Risk factors for these complications include a clinician with limited technical experience, the presence of emphysema, and a history of chest puncture. Hemothorax occurs in approximately 1.6% of thoracentesis procedures and is thus a rare complication [6]. In the present case, we observed no injury to the intercostal artery truncus but confirmed an abnormality in the course of the intercostal artery; therefore, we inferred that the cause of hemothorax in this case was injury to a small vessel, and not truncus due to the abnormality. In another case report similar to ours, a patient developed massive hemothorax due to an abnormality in the course of the intercostal artery [7]. The use of ultrasound guidance is considered useful to prevent injury to the intercostal artery. One study showed that the risk of bleeding associated with thoracentesis was 0.27 and 1.25% with and without ultrasound guidance, respectively [8]. Thus, it seems that the use of ultrasound will be indicated in the future.

## Conclusions

We have reported a case of hemorrhagic shock caused by vitamin K deficiency and an abnormality in the path of the intercostal artery. Even if platelet count is normal, measurement of coagulation function and supplementation of vitamin K are necessary in high-risk patients. Additionally, ultrasound guidance is useful during thoracentesis.

## Abbreviations

APTT: Activated partial thromboplastin time
PIVKA-II: Protein induced by vitamin K absence/antagonist-II
PT: Prothrombin time
PTGBD: Percutaneous transhepatic gallbladder drainage
SBT/ABPC: Sulbactam/ampicillin
